# Clinical and molecular characteristics of Chinese non‐small cell lung cancer patients with *ERBB2* transmembrane domain mutations

**DOI:** 10.1002/1878-0261.12733

**Published:** 2020-07-01

**Authors:** Yun Fan, Jinrong Qiu, Ruoying Yu, Ran Cao, Xiaoxi Chen, Qiuxiang Ou, Xue Wu, Yang W. Shao, Misako Nagasaka, Jiexia Zhang, Sai‐Hong Ignatius Ou

**Affiliations:** ^1^ Cancer Hospital of University of Chinese Academy of Sciences Zhejiang Cancer Hospital Hangzhou China; ^2^ Department of Biological Therapy Eastern Hepatobiliary Surgery Hospital Affiliated to Naval Military Medical University Shanghai China; ^3^ Translational Medicine Research Institute Geneseeq Technology Inc. Toronto Canada; ^4^ Nanjing Geneseeq Technology Inc. Nanjing China; ^5^ School of Public Health Nanjing Medical University Nanjing China; ^6^ Karmanos Cancer Institute Wayne State University Detroit MI USA; ^7^ State Key Laboratory of Respiratory Disease National Clinical Research Center for Respiratory Disease Guangzhou Institute of Respiratory Health The First Affiliated Hospital of Guangzhou Medical University China; ^8^ Chao Family Comprehensive Cancer Center University of California Irvine School of Medicine Orange CA USA

**Keywords:** comprehensive genomic profiling, *ERBB2*, NSCLC, TMD, transmembrane domain mutation

## Abstract

Transmembrane domain (TMD) mutations of *ERBB2* have previously been reported in lung cancer patients in addition to well‐studied kinase domain (KD) mutations, which may stabilize ERBB2 heterodimerization with other EGFR family members and favor a kinase active conformation. However, the frequency and clinical significance of *ERBB2* TMD mutations in Chinese population is unknown. We prospectively analyzed the next‐generation sequencing data of 34 368 Chinese lung cancer patients with different sample types, including tumor tissue, plasma, cerebrospinal fluid, and pleural effusion. Patients' clinical characteristics and treatment history were retrieved from the database for further evaluation. Our findings show that *ERBB2* V659/G660 mutations were detected at a frequency of 0.13% (45/34 368), of which the most frequent was V659D/E (88.9%), with a trend in nonsmokers and male. Moreover, 18% of patients (8/45) showed *EGFR* and/or *ERBB2* amplification, whereas nine patients presented *EGFR* L858R or exon19 deletion. Interestingly, novel *ERBB3* TMD mutation I646R was found coexisting in three patients with *ERBB2* V659D and one patient with *ERBB2* G660D, which might influence its heterodimerization with *ERBB2* and further activate *ERBB2*. Four *ERBB2* TMD mutation‐positive patients received afatinib monotherapy or combination therapy, but showed variable responses. One patient with V659E responded well to ERBB2 inhibitor lapatinib plus capecitabine as well as subsequent afatinib treatment upon progression. Our study provides valuable insights into the distribution of *ERBB2* TMD mutations by employing the largest Asian lung cancer cohort thus far. Patients with *ERBB2* TMD mutations who received afatinib, a pan‐ERBB inhibitor, demonstrated mixed responses, posing the urgent need to develop more effective therapeutic strategy for patients who carry *ERBB2* TMD mutations.

## Graphical abstract

Transmembrane domain (TMD) mutations of *ERBB2* were identified in 0.13% of a large Chinese lung cancer cohort with a ratio of 0.17% in lung adenocarcinomas. Novel *ERBB3* TMD mutation I646R was found co‐existing in four *ERBB2* TMD mutation carriers, which might affect ERBB2‐ERBB3 heterodimerization. *ERBB2* TMD carriers demonstrated mixed response to afatinib, posing the need for more effective therapeutic strategy.

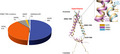

## 1 Introduction

Since the discovery of actionable activating epidermal growth factor receptor (*EGFR*) mutation and anaplastic lymphoma kinase (*ALK*) rearrangement in lung cancer, thoracic oncologists have led the way in comprehensive genomic profiling (CGP) of solid malignancies for precision medicine. Indeed, multiple additional actionable driver alterations have been identified including some of these mutations are at < 1% incidence in non‐small‐cell lung cancer (NSCLC) such as neurotrophic tropomyosin‐related kinase rearrangement (Gatalica *et al*., 2019) and *NRG1* fusions (Drilon *et al*., 2018). Another rare but potentially actionable driver mutation is located in the transmembrane domain (TMD) at valine amino acid residue 659 and glycine at amino acid residue 660 in Erb‐b2 receptor tyrosine kinase 29 (ERBB2) (Bargmann *et al*., 1986; Pahuja *et al*., 2018). Clinically, it was initially described in the index case of a family with familial lung cancer (Yamamoto *et al*., 2014). TMD mutations are typically mutually exclusive from HER2 kinase domain (KD) mutations and other oncogenic driver mutations. TMD mutations are located within the glycine zipper motif at the N‐terminal portion of TMD which is critically important to the dimerization of ERBB2 to other EGFR family members (Bocharov *et al*., 2008; Mineev *et al*., 2010). NMR studies of the protein revealed that TMD mutations stabilize the N‐terminal dimerization and favor a kinase active conformation (Ou *et al*., 2017). *In vitro* experiments confirmed that *ERBB2* TMD mutations can be inhibited by afatinib, a second‐generation EGFR/ERBB2 tyrosine kinase inhibitor (TKI), but not by gefitinib, a first‐generation EGFR TKI (Yamamoto *et al*., 2018). A subsequently larger survey of 8551 lung adenocarcinoma patients from a US‐based genomic profiling database revealed that *ERBB2* TMD mutations are very rare at a frequency of 0.18%. The study further suggested that *ERBB2* TMD mutant patients may respond to afatinib (Ou *et al*., 2017). In this study, considering the unique mutation spectrum of Chinese lung cancer patients compared to the western population, we investigated the frequency and mutation spectrum of *ERBB2* TMD mutations in Chinese lung cancer population, which would further our insights for treating *ERBB2* TMD mutant patients.

## 2 Material and methods

### 2.1 Patient cohort, sample preparation, and next‐generation sequencing

From January 2014 to July 2019, 34 368 unique cases of lung cancer were analyzed using CGP in a Clinical Laboratory Improvement Amendments‐certified, College of American Pathologists accredited laboratory (Nanjing Geneseeq Technology Inc., Nanjing, China), as previously described (Fang *et al*., 2019; Shu *et al*., 2017). Written consent was obtained from each patient. The study methodologies conformed to the standards set by the Declaration of Helsinki and were approved by the local ethics committee.

The pathologic diagnosis of each case was confirmed on routine hematoxylin and eosin (H&E) stained slides and all tumor samples forwarded for DNA extraction contained a minimum of 20% tumor nuclear area. DNA from formalin‐fixed paraffin‐embedded or fresh tumor samples, and/or cell‐free DNA (cfDNA) from plasma, cerebrospinal fluid (CSF), or pleural effusion (PE) were extracted as previously described (Jiang *et al*., 2017; Yang *et al*., 2018). The extracted DNA samples were assayed by next‐generation sequencing (NGS)‐based CGP through hybrid capture targeting all coding exons of solid tumor‐related genes and introns involved in cancer‐related gene fusions, including all EGFR family members. Captured libraries were sequenced using Illumina HiSeq platform (Illumina, San Diego, CA, USA) to a coverage depth of > 3000×, > 500×, > 100× for ctDNA, tissue and whole blood control samples, respectively. The resultant sequences were analyzed for base substitutions, insertions, deletions, copy number alterations, and gene fusions as previously described (Yang *et al*., 2018).

### 2.2 *In silico* modeling of *ERBB2‐ERBB3* TMD dimerization structure

The hetero‐dimerization structural model of *ERBB2–*Erb‐b2 receptor tyrosine kinase 3 (*ERBB3*) TMDs was built based on the NMR structure of *EGFR*‐TMD homo‐dimer (PDB ID: 2M20), which provided the structural basis for *EGFR* activation (Endres *et al*., 2013). The NMR structures of *ERBB2*‐TMD (PDB ID: 2N2A) and *ERBB3*‐TMD (PDB ID: 2L9U) were used individually in the superimpose. The built *ERBB2–ERBB3* TMDs model was further optimized in the structure to eliminate any existing steric clashes with ucsf PLOP6.0 software (Jacobson *et al*., 2002).

## 3 Results

### 3.1 *ERBB2* TMD mutations are rare in lung cancer, and majority are lung adenocarcinoma

**Fig. 1:**
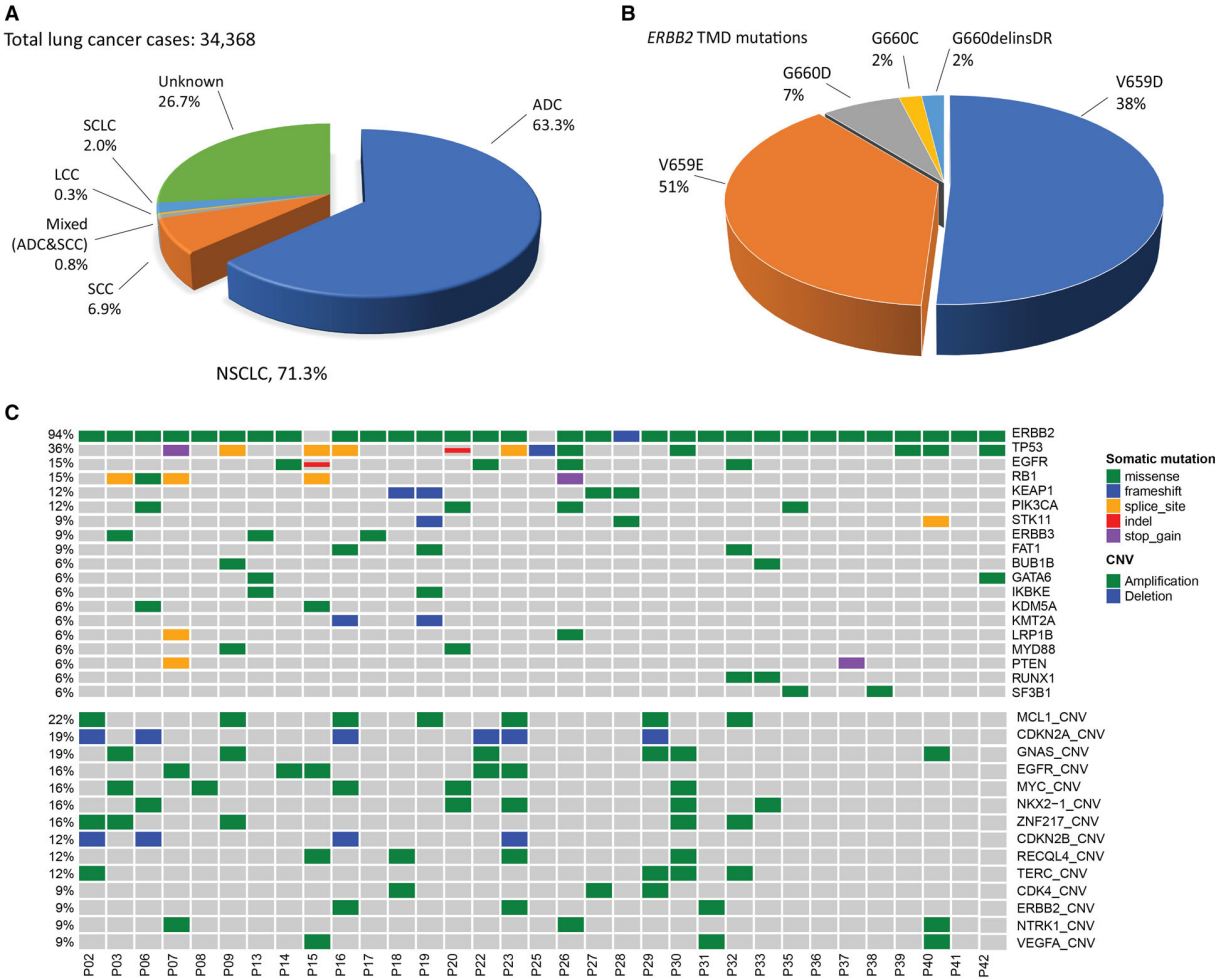
Genomic landscape of lung cancer patients with *ERBB2* TMD mutation. (A) Distribution of the histological subtypes of lung cancer cases analyzed. LCC, large‐cell carcinomas. (B) Distribution of *ERBB2/HER2* TMD mutations in a cohort of 45 patients with lung ADCs. (C) Mutation profile of lung cancer patients with *ERBB2/HER2* TMD mutations in tumor tissue. Each column represents one patient. Total number of mutation and amplification in each patient were shown in the top bar graph. Distributions of individual gene mutations (middle panel) and amplification (bottom panel) detected at least in two patients were shown.

Among the 34 368 unique clinical lung cancer cases evaluated with CGP in this study, 23 247 were NSCLC (71.3%), including 21 762 cases of adenocarcinoma (ADC, 63.3% of total), 2389 cases of squamous cell carcinoma (SCC, 6.9%), 260 cases of mixed ADC and SCC (mixed ADC & SCC, 0.8%), 97 cases of large cell carcinoma (0.3%), 681 cases of small cell carcinoma (SCLC, 2.0%), and 9179 cases of lung cancer not otherwise specified (Unknown; 26.7%; Fig. 1A).

The most common *ERBB2* mutations were the KD mutations at 2.3% (802 out of 34 368), followed by extracellular domain mutations (1.0%, 340 out of 34 368), which were identified in multiple subtypes of lung cancer. *ERBB2* TMD mutations were extremely rare at 0.13% (45 out of 34 368). Summary of the clinical characteristics of patients with *ERBB2* TMD mutation was shown in Table 1. Except six patients with unknown histology and one patient with mixed ADC and SCC, all patients with *ERBB2* TMD mutations were ADCs at a frequency of 0.17% (38 out of 21 762), consistent with the previous report in western lung cancer patients (Ou *et al*., 2017). There was slightly more male (51%) than female (45%) in patients with *ERBB2* TMD mutation. The median age of these patients was 59.5 ranging from 39 to 76. Of the thirteen patients with a known smoking history, nine were never‐smokers.

**Table 1:** Summary of clinical characteristics of *ERBB2/HER2* TMD mutant patients.

Characteristics	All patients (*N* = 45)
Median age (range)	59.5 (39–76)
Gender—No. (%)	
Male	23 (51)
Female	20 (45)
Unknown	2 (4)
Smoking—No. (%)	
Smokers	4 (9)
Never smokers	9 (20)
Unknown	32 (71)
Histology—No.(%)	
ADC	38 (85)
Mixed ADC and SCC	1 (2)
Unknown	6 (13)
Stage—No. (%)	
I	3 (7)
II	0 (0)
III	4 (9)
IV	16 (35)
Unknown	22 (49)
ERBB2 TMD mutation—No. (%)	
V659	40 (89)
G660	5 (11)

A comparison of *ERBB2* TMD mutation in a Western cohort and this Asian cohort was shown in Table 2, and distribution of different *ERBB2* TMD mutation was shown in Fig. 1B. 40 cases were found to harbor *ERBB2* TMD mutations at amino acid residue 659, including 23 cases of V659E and 17 cases of V659D. Only five cases with G660 TMD mutation were found in our cohort, including three cases of G660D mutation, one case of D660C and one case of G660delinsDR. Seven patients with *ERBB2* TMD mutation were found to be heterozygous for *BIM* deletion polymorphism.

**Table 2:** Comparison of *ERBB2/HER2* TMD mutations between US and Chinese NSCLC patients.

	USA	Chinese
Cohort size	15	45
Median age	54	59.5
Female gender	73.3%	45%
Specific mutation (%)		
V659E	9 (60.0)	23 (51.1)
V659D	3 (20.0)	17 (37.8)
G660D	2 (13.3)	3 (6.7)
G660C	0 (0.0)	1 (2.2)
G660R	1 (6.7)	0 (0.0)
G660insDR	0 (0.0)	1 (2.2)
V659_600VE	1 (6.7)	0 (0.0)
Concurrent alterations (%)		
*ERBB2* amplification	2 (13.3)	4 (8.9)
*EGFR* amplification		5 (11.1)
BIM deletion polymorphism		
Heterozygous		7 (15.6)

### 3.2 Concomitant *EGFR* family gene amplifications or activating mutations exist in *ERBB2* TMD mutant patients

In this cohort, patients had multiple sample types for NGS analysis including plasma (15), tumor tissue (34), and CSF (four). Two tumor samples were excluded from further analysis because they were sequenced with a different target gene panel (Fig. S1). Mutational landscape of patients with *ERBB2* TMD mutation in tumor tissue was shown in Fig. 1C. Identified gene alterations in plasma and CSF were shown in Table S1. Apart from the *ERBB2* mutation (94%, 30 out of 32), *TP53* alterations showed the highest ratio (38%, 12 out of 32) among 32 cases with *ERBB2* TMD mutation (Fig. 1C). In P15 and P25, *ERBB2* TMD mutation was identified in CSF and/or plasma but not in tumor tissue.

*EGFR* mutations were identified and accounted for 20% (nine out of 45) of all cases (Fig. 1C and Table S1). The activating *EGFR* mutation L858R was identified in tumor tissue of two patients with either *ERBB2* G660C (P22) or G660D (P26). In plasma and CSF, *EGFR* mutation L858R was identified in four patients (two *ERBB2* V659D and two *ERBB2* G660D) including P26 (Table S1). Patient 14 had two tumor tissue from the right upper and lower lobe of the lung, respectively. One lobe harbored *EGFR* amplification, while the other lobe was identified with *ERBB2* V596D and *EGFR* sensitizing mutation G719A of which occurs at ~ 2–3% in E*GFR*‐mutated lung tumors (Mitsudomi and Yatabe, 2010). Patient 15 with *ERBB2* TMD V659D detected both in plasma and in CSF had concurrent *EGFR* amplification and *EGFR* exon19 deletion. Patient 32 carried V659E TMD mutation and was also detected to carry the extracellular *EGFR* missense mutation A289V which has been previously reported as an oncogenic mutation in glioblastoma (Dai *et al*., 2018; Thorne *et al*., 2017). Patient 42 with *ERBB2* V659D had a *ERBB2* extracellular domain mutation S310F.

Myeloid leukemia cell differentiation protein 1 (22%) was the most frequent amplification found among *ERBB2* TMD carriers. *EGFR* amplification was identified in five patients with *ERBB2* TMD mutations, and *ERBB2* amplification was in four patients (Fig. 1C and Table S1). P23 with *EGFR* and *ERBB2* double amplification was also identified with fourteen gene amplifications which may be due to the chromosome instability (Fig. 1C).

### 3.3 Recurrent *ERBB3* TMD mutation in patients with *ERBB2* TMD mutation may affect heterodimerization between* ERBB2* and *ERBB3*

Concurrent *ERBB3* mutation was also identified among *ERBB2* TMD carriers. A novel *ERBB3* TMD mutation I649R was found in tumor tissue of three patients with *ERBB2* V659D (P03, P13, and P17). P24 with *ERBB2* G66D had *ERBB3* I649R detected in CSF (Table S1). Meanwhile, no other known driver mutation was detected in the four patients. Especially in P17 and P24, apart from *ERBB2* and *ERBB3* TMD mutations, no other gene alteration was identified in all the samples.

**Fig. 2:**
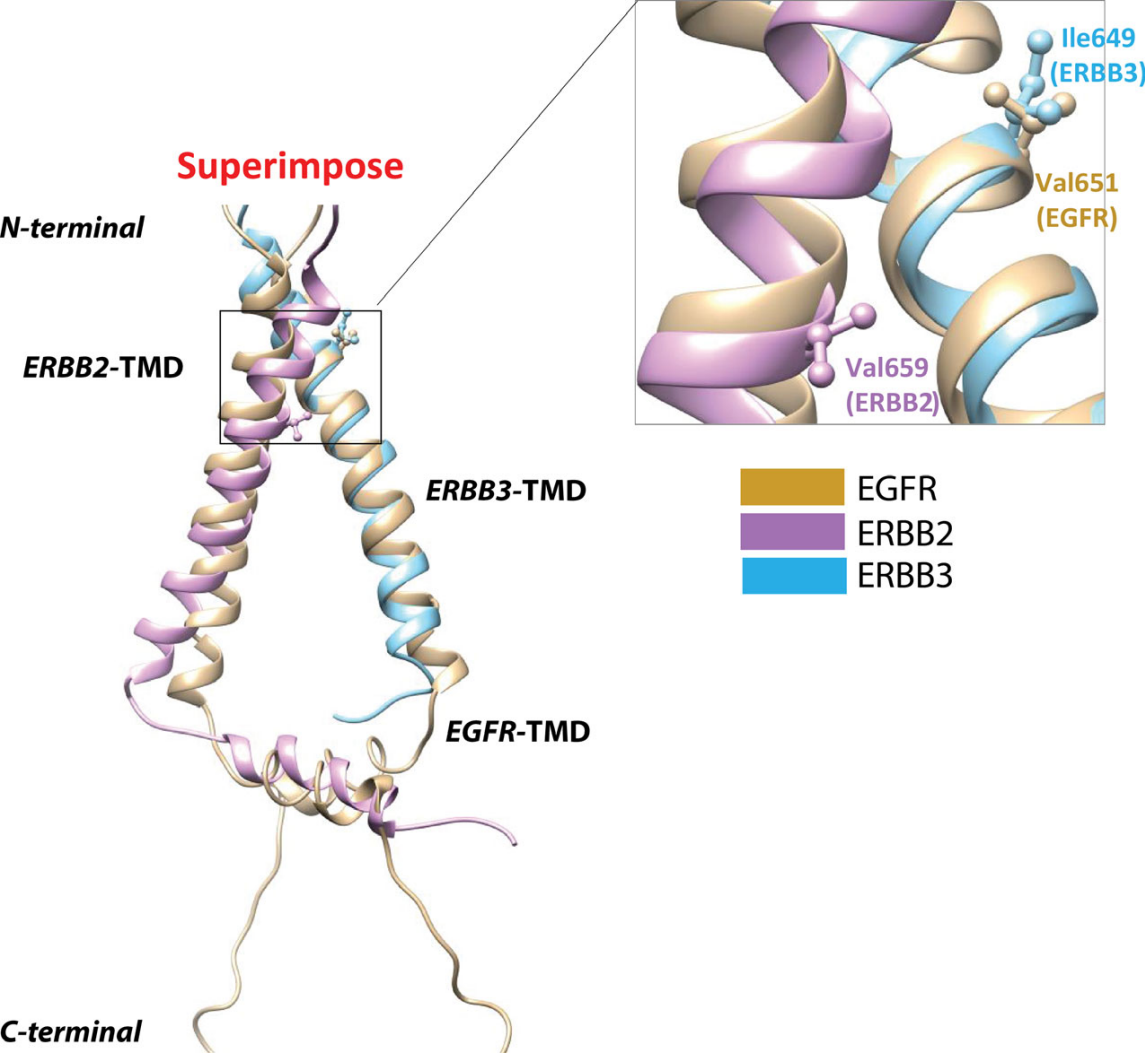
Structural analysis of influence of concurrent *ERBB2–ERBB3* TMD mutations on hetero‐dimerization. Superimpose of *EGFR*‐TMD homo‐dimer structure (PDB ID: 2M20, tan color) and modeled *ERBB2–ERBB3* TMD hetero‐dimer structure (HER2‐TMD PDB ID: 2N2A, magenta color; ERBB3‐TMD PDB ID: 2L9U, cyan color). The highlighted residues including Val659 in *ERBB2*, Ile649 in *ERBB3*, and Val651 in *EGFR* are shown in stick‐and‐ball. Left panel: general view of the whole structure of TMD. Right panel: focused view of the interested region bearing somatic mutations.

Activation mechanism underlying the hetero‐dimerization of *ERBB2*/*ERBB3* TMDs was still unknown despite the well‐established hetero‐dimerization activation mechanism of KD. Comparison of *in silico* model of *ERBB2‐ERBB3* TMD hetero‐dimer and experimental structure of *EGFR* TMD homo‐dimer showed that *ERBB3*‐Ile649 was at the same location to *EGFR*‐Val651 in the TMD region (Fig. 2), whose polar mutation was predicted to enhance TMD dimerization and activating the receptor. In addition, I649R in ERBB3 greatly increased the hydrophilic surface of the N‐terminal dimerization motif as well as its polarity, which had the potential to facilitate transition of receptor dimer to an active state independently of ligand binding (Notsuda *et al*., 2017). It was highly likely that I649 on *ERBB3* could enhance TMD heterodimerization and activating *ERBB2*.

### 3.4 Treatment response of patients with *ERBB2* TMD mutation

**Fig. 3:**
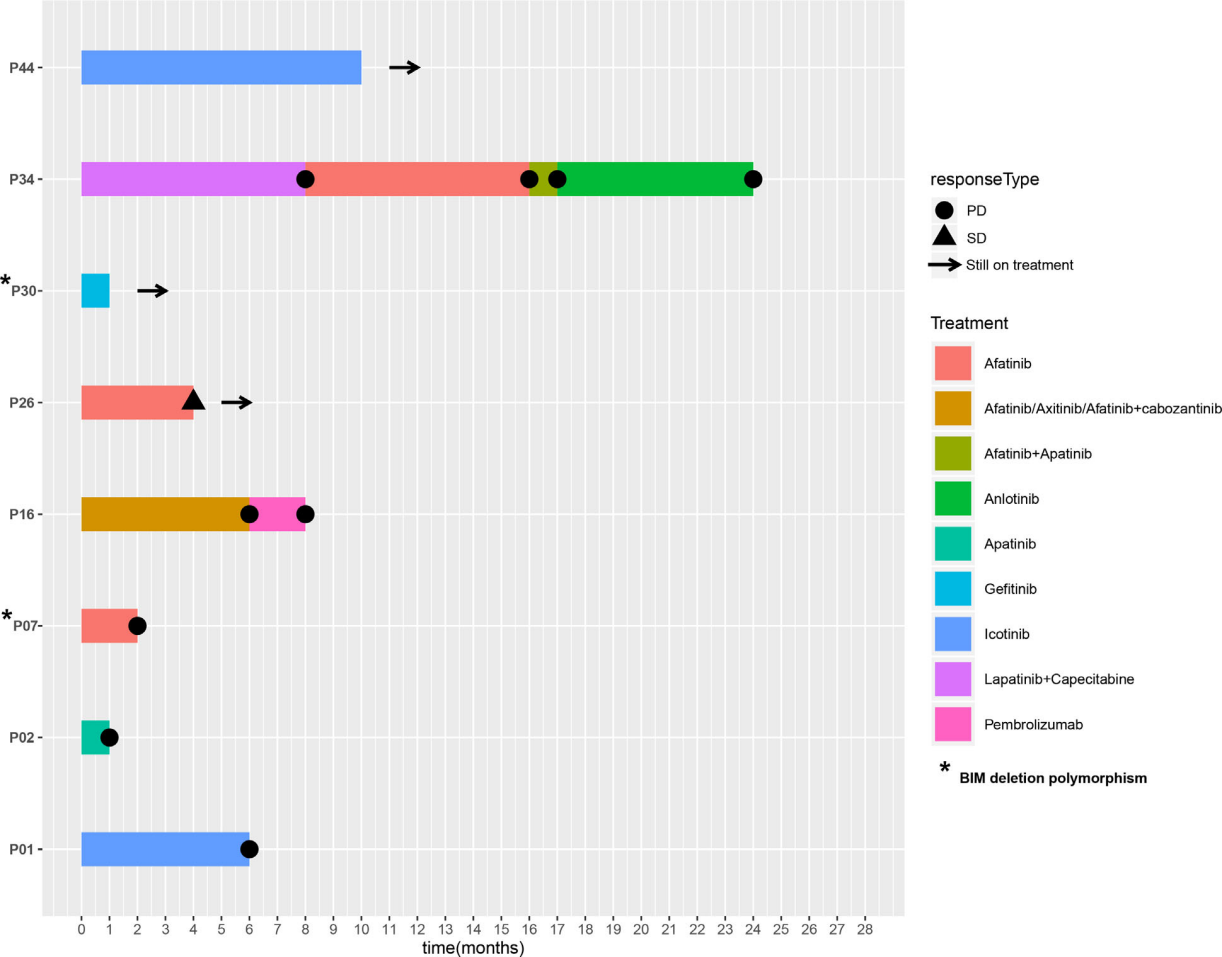
Treatment outcomes of different target therapy in patients with *ERBB2* TMD mutations. The horizontal axis shows the duration (months) since initiation of TKI treatment. The drugs used in each treatment were indicated by different colors. /: sequential treatment; +: combined treatment.

The treatment history and clinical outcomes of target therapy were available for a total of eight patients (Fig. 3). Target therapy with tyrosine kinase inhibitor (TKI) alone or combination therapy was administrated with variable responses. Two patients (P01 and P44) were administrated with icotinib, a first‐generation EGFR TKI. Patient 1 (V659E) reached a PFS of 6 months, while patient 44 (G660D) was still on treatment after 9 months. No *EGFR* alteration was detected in P01. P44 was identified with *EGFR* L858R in the plasma.

Four patients were administrated with afatinib which is TKI target both *EGFR* and *ERBB2*. Patient 7 had *ERBB2* V659E mutation as well as *EGFR* amplification. He was treated with afatinib with rapid disease progression within 2 months. The patient in case 16 (V659E) was a 39‐year‐old female diagnosed as lung ADC. She achieved no response to the 6‐month sequential treatment of afatinib, axitinib, and afatinib plus cabozantinib. Then, she underwent immunotherapy with pembrolizumab, a PD‐1 inhibitor, and had progressive disease (PD) after 2 months of treatment. Patient 26 carrying both *ERBB2* G660D and *EGFR* L858R mutation received afatinib treatment and achieved stable disease (SD) for only 4 months, which may have been due to a concurrent *PIK3CA* E545K mutation that can influence the efficacy of TKI treatment. Indeed, a follow‐up NGS testing at disease progression revealed a dramatic increase in the mutant allele frequencies of all three mutations. Interestingly, patient 34 harboring *ERBB2* V659E was given *ERBB2* TKI lapatinib plus capecitabine with a progression‐free survival (PFS) of 8 months. He then switched to afatinib treatment and further achieved a PFS of 8 months, although the subsequent afatinib plus apatinib treatment and anlotinib therapy were not effective upon disease progression.

## 4 Discussion

In this study, we reported on the clinical and molecular characteristics of *ERBB2* TMD in Asian lung cancer patients. Our analysis revealed that *ERBB2* TMD mutation accounted for 0.13% of all lung cancer cases, which was extremely rare and majority were restricted to lung ADC. This observation also suggested a similar frequency of TMD mutation ratio in ADC (0.17%, 38 out of 21 762). Besides the previously reported V659D/E and G660D mutations, we also observed novel *ERBB2* TMD mutation G660C and G660delinsDR in our cohort.

Here, the incidence of ERBB2 TMD mutation was analyzed with different sample type including plasma, tissue, and CSF. Given this was a retrospective analysis of a large Asian NSCLC cohort, the inconsistency in sample type was quite common in this real‐world situation. Tumor tissues could only be obtained when patients underwent surgery. And in some patients, only the non‐invasive liquid biopsies were performed. Generally, positive rate of NGS detection in plasma, tissue, and CSF is 75%, 93%, and 82%, respectively, calculating with all NGS data from 34 368 Chinese lung cancer patients. This difference in sample type and sensitivity of NGS detection might have impact on the interpretation on *ERBB2* TMD mutation prevalence and concurrent genomic alterations, which could be considered as a major limitation of this study.

Afatinib has demonstrated response in patients with *ERBB2* TMD mutation in other study (Ou *et al*., 2017). In this cohort, two patients achieved PD and one patient achieved SD on afatinib treatment. Despite coexistence of other driver event which may impact the efficacy of afatinib, another reason for the inferior response to afatinib may be the *BIM* deletion polymorphism. *BIM* is a proapoptotic member of the B‐cell CLL/lymphoma 2 (BCL2) family of proteins, and its upregulation is required for TKIs to induce apoptosis in kinase‐driven cancers. It has been reported that *EGFR*‐mutated NSCLC patients with *BIM* polymorphism experienced significantly worse responses to TKIs than patients without this polymorphism (Ng *et al*., 2012). Further investigation needs to be carried out on the association of *BIM* polymorphism and clinical outcomes of *ERBB2*‐mutated NSCLC patients under TKI treatment.

Regarding concurrent *ERBB3* TMD mutation at I649, previous reports have shown that *ERBB3* mutations alone are not sufficient to result in oncogenic transformation (Jaiswal *et al*., 2013). Notably, recurrent of *ERBB3–ERBB2* TMD double mutations in *ERBB2* TMD carriers and structure analysis suggested that *ERBB3* I649R may be a aid in driving the oncogenicity of *ERBB2*. Further study is warranted to test this hypothesis. Similar to V659/G660 of ERBB2, V651/G652 of EGFR was predicted to act in the same fashion by enhancing TMD dimerization in certain conformation (Ou *et al*., 2017). Interestingly, we also identified seven patients with the *EGFR* TMD mutation (three with *EGFR* TMD V651M, two with *EGFR* TMD V651L, one with *EGFR* TMD G652D, and one with *EGFR* TMD G652W; Table S2). The four patients with *EGFR* TMD mutation at V651 carried concurrent *EGFR* classic oncogenic mutation L858R or exon19 deletion which suggested that these *EGFR* TMD mutations may not necessarily be the driver mutation in these patients.

## 5 Conclusion

Our study provided valuable insights into the distribution of *ERBB2* TMD mutations by employing the largest Asian lung cancer cohort thus far. Our findings showed that *ERBB2* V659/G660 mutations were detected in 0.17% of lung ADC cases and 0.4% (one out of 260) of cases with mixed ADC and SCC. Patients with *ERBB2* TMD mutations who received afatinib—a pan‐ERBB inhibitor demonstrated mixed responses, posing the urgent need to develop more effective therapeutic strategy for patients who carry *ERBB2* TMD mutations.

## 6 Implication for practice

- *ERBB2* TMD alterations are presented in 0.17% of Chinese lung ADC patients.
- Eighteen percent of the patients who were identified with *ERBB2* TMD mutations had concurrent *EGFR* mutations or *EGFR/ERBB2* amplification.
- A novel *ERBB3* TMD mutation I646R was found coexisting in four patients with *ERBB2* TMD mutation, which might affect its ERBB2‐ERBB3 heterodimerization and activate ERBB2.
- Patients with *ERBB2* TMD mutations who received afatinib—a pan‐ERBB inhibitor—demonstrated mixed responses. More effective treatment options are in urgent need for patients who carry *ERBB2* TMD mutations.

## Conflict of interest

RY, XW, RC, XC, and QO are the employees of Geneseeq Technology Inc., Canada. YWS is an employee and shareholder of Nanjing Geneseeq Technology Inc., Nanjing, Jiangsu, China. MN received honorarium from Astra Zeneca and Tempus. SHIO has received speaking/advisory honorarium from Pfizer, Merck, Roche/Genentech, Takea/ARIAD, and AstraZeneca. SHIO is a stock owner and former member of the scientific advisory board of Turning Point Therapeutics, Inc. The remaining authors have no conflict of interest to declare.

## Author contributions

YF and JQ involved in conception and design. YF, JQ, and JZ carried out provision of study material or patients. RY, XW, and QO involved in collection and/or assembly of data. RY, XW, QO, YWS, XC, and RC analyzed and interpreted the data. SHIO, JZ, and MN wrote the manuscript. SHIO involved in final approval of manuscript.

[Correction added on 11 July 2020, after first online publication: the author name Xiaoxi Chen(XC) has been included in Conflict of interest and Author contributions sections]

## Supporting information


**Fig. S1.** Consort diagram of sample inclusion criteria.

**Table S1.** Gene alterations identified in plasma and CSF of *HER2* TMD mutant patients.

**Table S2.** Clinicopathologic features of *EGFR* TMD mutations in lung cancer patients.
