# Supplementary figures and images for: Clinical and molecular characteristics of Chinese non‐small cell lung cancer patients with ERBB2 transmembrane domain mutations
Source: Mol Oncol. 2020 Jul 1;14(8):1731–9. doi: 10.1002/1878-0261.12733 (PMC7400783; doi:10.1002/1878-0261.12733)

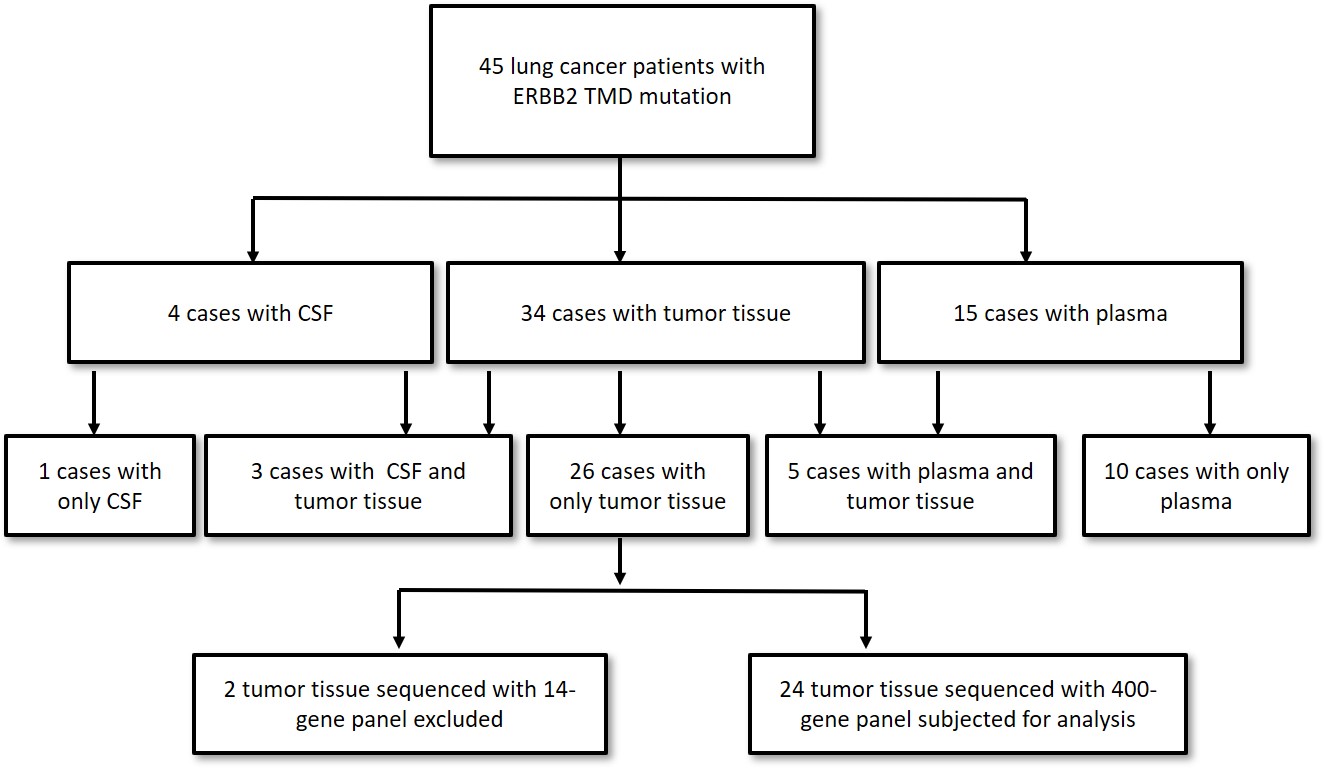

Supplement: Supplementary file 1 — Fig. S1. Consort diagram of sample inclusion criteria. [file MOL2-14-1731-s001.jpg]
