# Supplementary material for: Clinical and molecular characteristics of Chinese non‐small cell lung cancer patients with ERBB2 transmembrane domain mutations
Source: Mol Oncol. 2020 Jul 1;14(8):1731–9. doi: 10.1002/1878-0261.12733 (PMC7400783; doi:10.1002/1878-0261.12733)
Supplement: Supplementary file 3 — Table S2. Clinicopathologic features of EGFR TMD mutations in lung cancer patients. [file MOL2-14-1731-s003.docx]

**Supplementary Table 2.** Clinicopathologic features of *EGFR* TMD mutations in lung cancer patients.

| Patient ID | Sex | Age at diagnosis | Smoking history | Histology type | Diagnose Stage | EGFR TMD mutation | Concurrent ERBB family alterations | Treatment History |
| --- | --- | --- | --- | --- | --- | --- | --- | --- |
| P01 | F | 67 | Never-smoker | ADC | III | V651M | EGFR L747_T751del | NA |
| P02 | F | 41 | NA | ADC | IV | V651M | EGFR L858R | NA |
| P03 | NA | 49 | NA | ADC | NA | V651M | EGFR L858R | Surgery; Chemotherapy |
| P04 | F | NA | NA | NA | NA | V651L | No | NA |
| P05 | F | 79 | NA | ADC | IV | V651L | EGFR L858R | NA |
| P06 | M | 82 | NA | NA | IV | G652W | ERBB4 R72W | NA |
| P07 | F | 73 | NA | ADC | IV | G652D | EGFR Y299_V300delinsW | NA |

NA: not available
